# Asthma diagnosis and treatment – 1029. Yoga as an adjuvant therapy in asthma management

**DOI:** 10.1186/1939-4551-6-S1-P28

**Published:** 2013-04-23

**Authors:** Surya Kant, Shruti Agnihotri

**Affiliations:** 1 Pulmonary Medicine, King George's Medical University, Lucknow, India

## 

About 300 million people are suffering from asthma globally and about 10% of it about 30 million asthmatics belong to India. Current treatment involves use of preventer and reliever inhalers, antileukotrines and methyl xanthines. Many studies have shown the effect of yoga on bronchial asthma as significant improvement in pulmonary functions, quality of life and decrease in medication use but only a few studies have attempted to show the effect of yoga on bio- chemical changes.

The present study was conducted on 276 subjects with mild or moderate bronchial asthma who were allocated randomly to either the cases/ yoga (intervention) group (n= 121) and the control group (n= 120) in the Department of Pulmonary Medicine, King George’s Medical University, Lucknow, U.P. The yoga group received an intervention based on yoga (asanas, pranayama & meditation), in addition to standard medical treatment and the control group received only standard medical treatment and both groups were assessed at 0^th^, 3^rd^ and 6^th^ month.

There was a significant improvement found in asthma symptom score and Asthma Quality of Life scores in yoga group than control group. There was significant improvement found in day time symptoms in yoga group at 3^rd^ month & 6^th^ month than control group. Significant improvement was found in yoga group at 3^rd^ month (p- value 0.004) & 6^th^ month (p- value < 0.0001) in total AQOL score and its sub- domains (activity limitation (p- value <0.0001) & emotional function (p- value 0.006 & < 0.0001) in comparison to control group. In the yoga group, there was a steady and progressive improvement found in pulmonary functions as compared to the control group. There was significant difference found in haemoglobin, TLC, Eosinophils, Monocytes & Super oxide dismutase level. The rescue medication use has a significant decreased in comparison to control group.

The present study showed that yoga group has significant improvement in asthma symptom scores, spirometrical, bio- chemical changes, quality of life and also helps in reduction of inhalation therapy, thus reducing the cost of therapy. So, it can be useful cost effective add on therapy of asthmatics.

